# Impact of relaxing incisions on maxillofacial growth following Sommerlad–Furlow modified technique in patients with isolated cleft palate: a preliminary comparative study

**DOI:** 10.1186/s12893-023-02247-5

**Published:** 2023-11-23

**Authors:** Sadam Ahmed Elayah, Min Wu, Essam Ahmed Al-Moraissi, Jiayi Yin, Karim Ahmed Sakran, Waseem Saleh Al-Gumaei, Hamza Younis, Ibtehal Almagrami, Nadia E. Alqadasy, Yang Li, Bing Shi

**Affiliations:** 1 State Key Laboratory of Oral Diseases & National Center for Stomatology &, National Clinical Research Center for Oral Diseases and Department of Oral and Maxillofacial Surgery, West China Hospital of Stomatology, Sichuan University, Chengdu, 610041 Sichuan China; 2 Department of Oral and Maxillofacial Surgery, College of Dentistry, Ibb University, Ibb, Yemen; 3 Department of Oral and Maxillofacial Surgery, College of Dentistry, Thamar University, Thamar, Yemen; 4 State Key Laboratory of Oral Diseases and National Clinical Research Center for Oral Diseases and Department of Orthodontics, West China Hospital of Stomatology, Sichuan University, Chengdu, China; 5 Department of Orthodontics, Faculty of Dentistry, First Affiliated Hospital of Zhengzhou University, Henan, China; 6 Department of Orthodontics, College of Dentistry, Ibn Al-Nafis University for Medical Sciences, Sana’a, Yemen

**Keywords:** Relaxing incisions, Cleft palate, Palatoplasty, Maxillofacial growth

## Abstract

**Objective:**

To estimate the impact of relaxing incisions on maxillofacial growth following Sommerlad-Furlow modified technique in patients with isolated cleft palate.

**Study design:**

A Retrospective Cohort Study.

**Methods:**

A total of 90 participants, 60 patients with non-syndromic isolated soft and hard cleft palate underwent primary palatoplasty (30 patients received the Sommerlad-Furlow modified technique without relaxing incision (S.F^−RI^ group), and 30 received Sommerlad-Furlow modified technique with relaxing (S.F^+RI^ group) with no significant difference found between them regarding the cleft type, cleft width, and age at repair. While the other 30 were healthy noncleft participants with skeletal class I pattern as a Control group. The control group (C group) was matched with the patient groups in number, age, and sex. All participants had lateral cephalometric radiographs at least 5 years old age. The lateral cephalometric radiographs were taken with the same equipment by the same experienced radiologist while the participants were in centric occlusion and a standardized upright position, with the transporionic axis and Frankfort horizontal plane parallel to the surface of the floor. A well-trained assessor (S. Elayah) used DOLPHIN Imaging Software to trace twice to eliminate measurement errors. All the study variables were measured using stable landmarks, including 12 linear and 10 angular variants.

**Results:**

The mean age at collection of cephalograms was 6.03 ± 0.80 in the S.F^+RI^ group, 5.96 ± 0.76 in the S.F^−RI^ group, and 5.91 ± 0.87 in the C group.

Regarding cranial base, the results showed no statistically significant differences between the three groups in S–N and S–N-Ba. While the S.F^+R.I^ group had a significantly shortest S-Ba than the S.F^−R.I^ & C groups (*P* = 0.01 & *P* < 0.01), but there was no statistically significant difference between S.F^−R.I^ & C groups (*P* = 0.71).

Regarding the skeletal maxilla, there was no significant difference between the S.F^+R.I^ and S.F^−R.I^ groups in all linear measurements (N-ANS and S-PM) except Co-A, the S.F^+R.I^ group had significantly shorter Co-A than the S.F^−R.I^ & C groups (*P* =  < 0.01). While the angular measurement, S.F^+R.I^ group had significantly less SNA angle than the S.F^−R.I^ & C groups (*P* =  < 0.01).

Regarding mandibular bone, there were no statistically significant differences in all linear and angular mandibular measurements between the S.F^+R.I^ and S.F^−R.I.^groups.

Regarding intermaxillary relation, the S.F^+R.I^ group had significant differences in Co-Gn—Co-A and ANB compared to the S.F^−R.I^ & C groups (*P* =  < 0.01). While there was no statistically significant difference in PP-MP between the three groups.

**Conclusion:**

As a preliminary report, the Sommerlad-Furlow modified technique without relaxing incisions was found to have a good maxillary positioning in the face and a satisfactory intermaxillary relationship compared to the Sommerlad-Furlow modified technique with relaxing incisions.

**Supplementary Information:**

The online version contains supplementary material available at 10.1186/s12893-023-02247-5.

## Introduction

Palatoplasty has advanced beyond just closing the gap to properly functioning palate reconstruction with minimal influence on maxillofacial growth in recent years [1]. The ideal surgical outcomes of a palate repair should include disconnection of the oral and nasal cavities and competent velopharyngeal closing for speech recovery while maintaining the normal potential growth in the relevant region [2]. The cause of restricted maxillary growth in individuals with a cleft palate following their initial palate repair surgery remains a topic of ongoing debate, as no agreement has been reached thus far. Moreover, there is a scarcity of compelling evidence establishing a link between growth restriction and the numerous possible factors that may contribute to this condition [3–5].

There is limited evidence indicating that the utilization of surgical relaxing incisions during the during primary palatoplasty can have a notably negative impact on the growth of the maxilla [4, 6]. Maxillofacial growth was reported to be inhibited following V–Y pushback and von Langenbeck approaches [7, 8], and denuded areas of bone subsequent relaxing incision left for secondary intention healing is mainly considered responsible for the disturbance of ensuing growth [9–13]. Numerous experimental studies have provided compelling evidence indicating that maxillary growth is adversely affected when the palatal bone is surgically removed using a relaxing incision. Techniques that involve minimal areas of denuded palatal bone are less likely to have negative effects on the maxillary growth when compared to other techniques with relaxing incisions [14–16].

The formation of scar tissue within the denuded palatal bone, subsequent to its development, is believed to be a contributing factor to maxillary dysgenesis [17]. Therefore, in a functional cleft palate repair, there has been a trend towards focusing more on palatoplasty techniques that avoid relaxing incisions on the hard palate [18, 19].

Conversely, some previous studies have concluded that there was no observed correlation between the utilization of relaxing incisions and the maxillary growth impairment [20, 21]. Thus, the impact of relaxing incisions on maxillofacial growth during palatoplasty remains a topic of debate and further research is needed to fully understand its effects [20, 22].

Sommerlad–Furlow modified technique (S-F technique) is a surgical technique developed by the author (S.B.), combines the best aspects of the Sommerlad approach (involving careful dissection of the muscles) and the Furlow approach (employing a Z-plasty technique) [23, 24]. Thus, S-F technique presents a commendable model for exploring the correlation between relaxing incisions and the maxillofacial growth.

The purpose of this study was to estimate the impact of relaxing incisions on maxillofacial growth following S-F technique in patients with isolated cleft palate.

## Materials and methods

### Subjects

A retrospective study was conducted on 90 participants, 60 patients with non-syndromic isolated soft and hard cleft palate (ISHCP) who underwent to Sommerlad–Furlow modified (S.F) technique, 30 patients received S.F without relaxing incision (S.F^−R. I^ group) 30 patients received S.F with relaxing incision (S.F^+R.I^ group) during the period from 2015 to 2018. While the other 30 were healthy noncleft participants with skeletal class I pattern (C group). Both palatoplasty techniques were performed by two highly experienced cleft surgeons who were trained by the same surgeon, Shi Bing. These surgeons worked as a team at the West China Stomatology Hospital, Sichuan University. Due to differences in etiology and morphology of cleft lip and palate (CLP) and isolated cleft palate (ICP) vary significantly, it is important to avoid combining patients with ICP and CLP in research studies. Therefore, when conducting scientific studies involving individuals affected by clefts, it is crucial to analyze these two groups separately [25, 26]. Furthermore, it is worth noting that the racial aspect can exert a noteworthy influence on the process of cleft palate repair [27]. As a result, numerous studies can be found comparing patients with clefts, without non-cleft control groups sharing the same ethnic background [26, 28]. To be more specific and more accurate, our study was conducted patients with the same cleft palate type; Veau II (involves the soft and hard palate but not alveolar process) [24, 29], as well as participants in all groups were from the same ethnicity. This study design is retrospective, which means that we collected and analyzed data from past records. Thus, authors did not decide which patient would receive palatoplasty with relaxing incisions and which would receive palatoplasty without relaxing incisions. Both patient groups were selected based on specific inclusion criteria: Han Chinese patients with nonsyndromic ISHCP who underwent S.F technique ( with/ without relaxing incisions), patients who had lateral cephalometric radiographs at least 5 years after palatoplasty, patients who had not undergone any other surgery besides palatoplasty as Cheiloplasty, Rhinoplasty or Orthodontic treatment, no history of other types of congenital malformation, there were no significant differences between S.F^+R.I^ group and S.F^−R.I^ group in gender, cleft width, age at the palatoplasty and age at cephalograms collection (Table 1). The cleft width was measured clinically during primary repair while the patients were under general anesthesia using a simple ruler and a caliper, which is a common method in the relevant literature [30]. It was measured at the junction of the hard and soft palate as the distance between the cleft margins [31]. The control group was matched with the patient groups in gender and age at cephalograms collection (Table 1). The study protocol was reviewed and approved by the Research Subject Review Board and Ethical Scientific Board of Sichuan University study (No. WCHS-CRSE-2023–113-R2-P). Informed consent was obtained from all their parents.

**Table 1:** Demographic features of participants of groups

Variables	S.F^+R.I^ group	S.F^−R.I^ group	Control group	*P-value*	Test type
**Gender**					
Male	14 (46.7%)	15 (50%)	16 (53.3%)	0.95	Chi-square test
Female	16 (53.3%)	15 (50%)	14 (46.7%)		
**Cleft width**, mm					
Mean ± SD	11.52 ± 2.18	10.92 ± 2.43	–	0.34	Independent t-test
(Min–Max)	(8–16)	(7–14)			
**Age at the palatoplasty**, year					
Mean ± SD	1.08 ± 0.33	1.04 ± 0.24	–	0.43	Independent t-test
(Min–Max)	(0.67–1.88)	(0.5–1.67)			
**Age at cephalograms collection**, year					
Mean ± SD	6.03 ± 0.80	5.96 ± 0.76	5.91 ± 0.87	0.83	ANOVA test
(Min–Max)	(5–7)	(5–7)	(5–7)		

*S.F*^*+R.I*^* group* Sommerlad-Furlow modified technique with relaxing incisions

*S.F*^*−R.I*^* group* Sommerlad-Furlow modified technique without relaxing incisions

*SD* Standard deviation

### Sample size calculation

The G*power 3.0.10 software was used to calculate the sample size. An effect size of 0.39 was obtained from a previous study [32] for the outcome of S–N between three groups after palatoplasty. The power of the study was set at 0.85, and the alpha error (*p*-value) was set at 0.05. Accordingly, the required sample size was 25 subjects for each group.

### Surgical technique

**Fig. 1:**
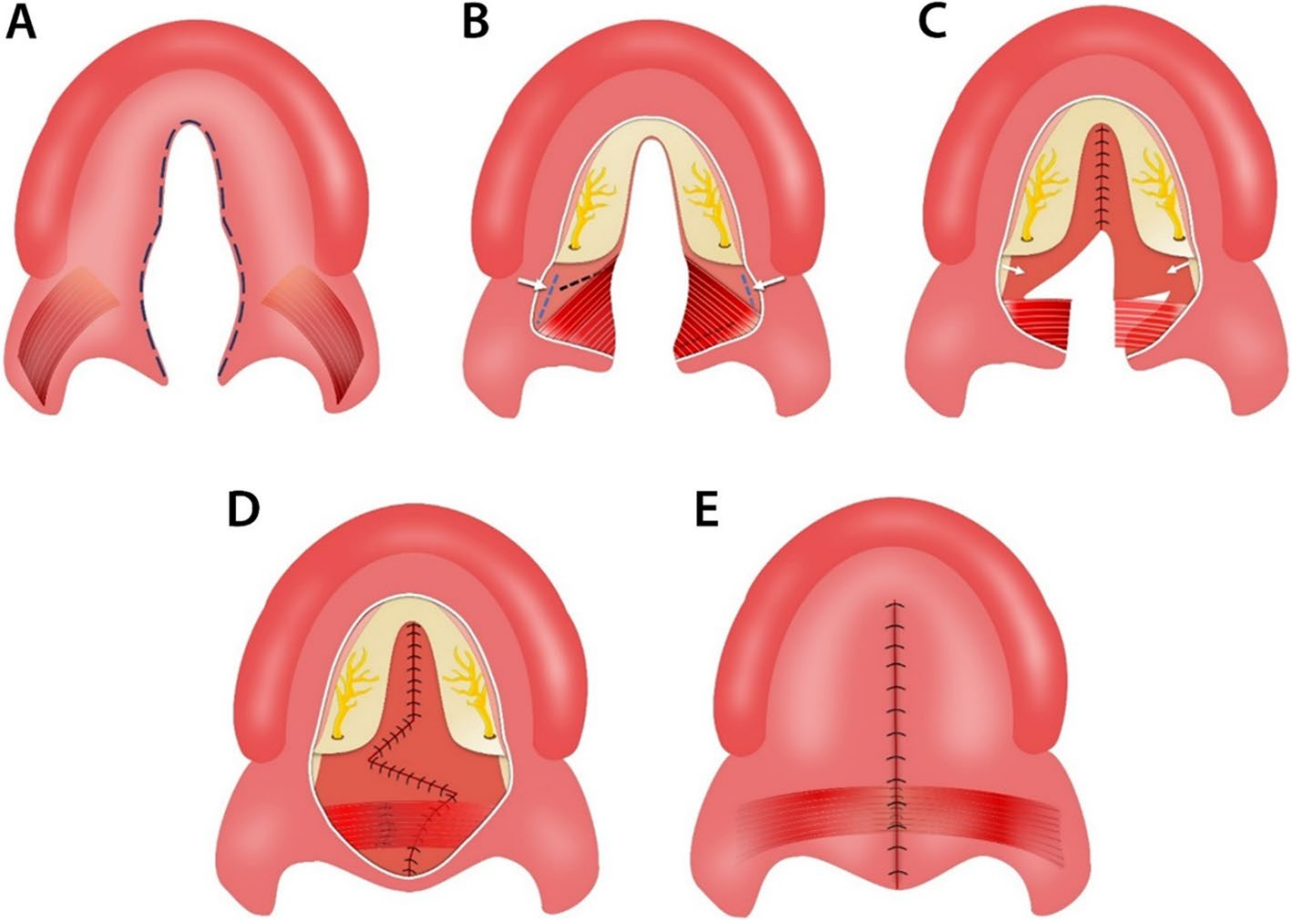
The surgical procedures of palatoplasty using the Sommerlad-Furlow modified technique without relaxing incisions. **A** an incision was made along the edge of the cleft to separate the oral mucosa layer and nasal mucosa layer. **B** A considerable amount of hard palate mucoperiosteal flap elevation and release of greater palatine neurovascular pedicles, nasopharyngeal incision is made on the medial pterygoid plate using an electrotome. **C** The nasal mucoperiosteum was peeled off anteriorly from the palatine bone and medially from the medial pterygoid plate toward the cranial base and suturing the nasal layer of the hard palate. The nasal musculomucosal layer was subjected to radical muscle dissection. then Z-plasty flaps on the nasal layer of the soft palate were designed. **D** Complete suturing of the nasal layer of soft palate then suturing the dissected palatal muscle. **E** The oral layer is sutured without relaxing incisions

**Fig. 2:**
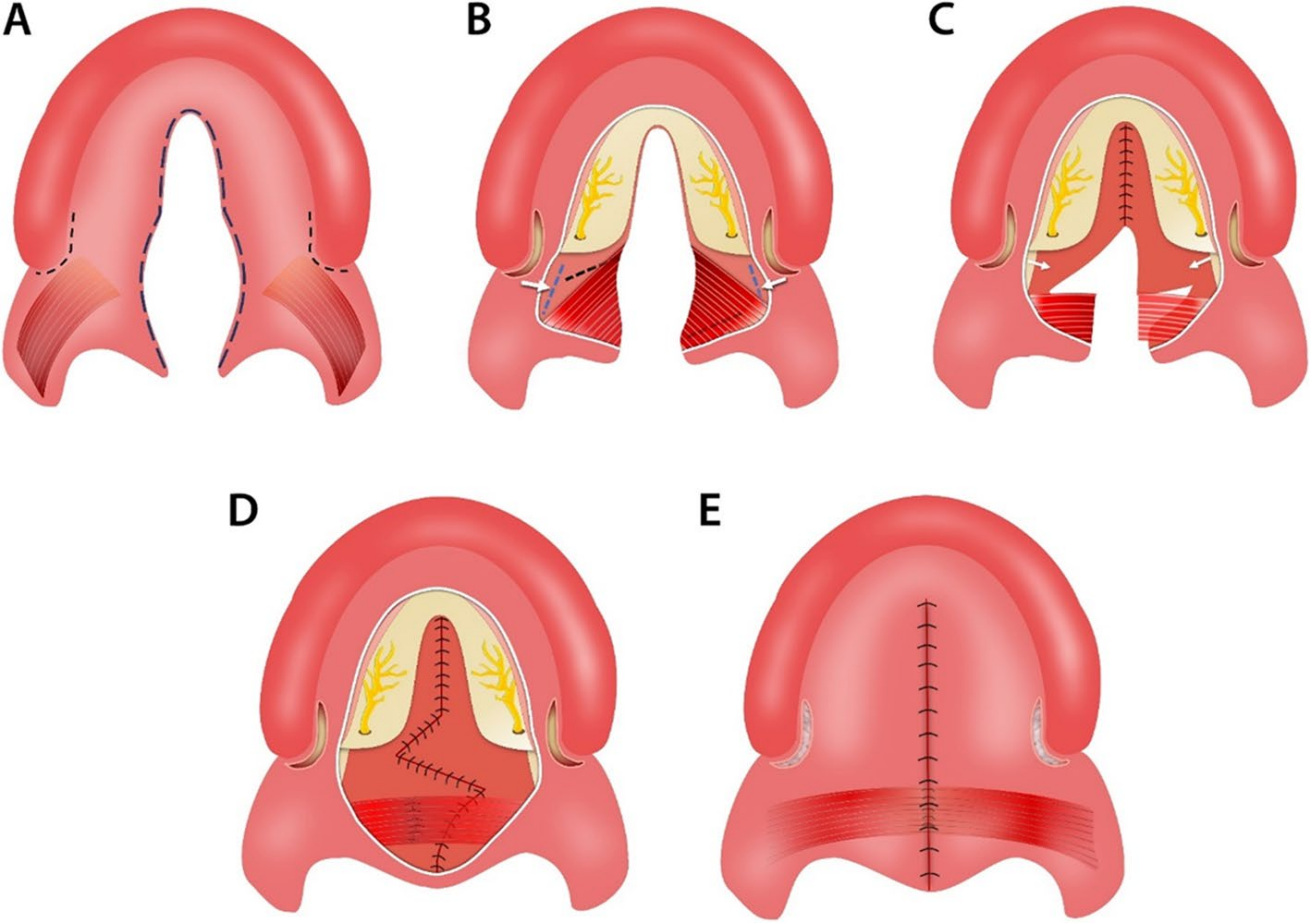
The surgical procedures of palatoplasty using the Sommerlad-Furlow modified technique with relaxing incisions. **A** an incision was made along the edge of the cleft to separate the oral mucosa layer and nasal mucosa layer and use of relaxing incisions on both cleft side. **B** A considerable amount of hard palate mucoperiosteal flap elevation and release of greater palatine neurovascular pedicles, nasopharyngeal incision is made on the medial pterygoid plate using an electrotome. **C** The nasal mucoperiosteum was peeled off anteriorly from the palatine bone and medially from the medial pterygoid plate toward the cranial base and suturing the nasal layer of the hard palate. The nasal musculomucosal layer was subjected to radical muscle dissection. then Z-plasty flaps on the nasal layer of the soft palate were designed. **D** Complete suturing of the nasal layer of soft palate then suturing the dissected palatal muscle. **E** The oral layer is sutured and fixing relaxing incisions with absorbable hemostatic sponge

Every patient in this study underwent cleft palate repair using S-F technique with or without relaxing incisions. The main points of the S-F technique can be summarized as follows [24, 33]: Initially, the procedure involves making an incision at the junction border of oral and nasal layers, followed by lifting the oral muco-periosteal flaps on the hard palate and freeing the greater palatine neurovascular pedicles. Subsequently, an incision is created in the nasopharynx on the medial pterygoid plate using an electrotome, and the nasal mucoperiosteum is cautiously separated from the plate. A meticulous dissection of the nasal musculomucosal layer is then carried out on the left side (mainly the levator veli palatini), and Z-plasty flaps are designed on the nasal layer. The dissected muscular flap from the palate is subsequently stitched to the myomucosal flap on the right side, and finally, the oral layer is closed with or without relaxing incisions [24] (Figs. 1 and 2).

### Cephalometric assessment

All of the lateral cephalometric radiographs were taken with the same equipment by the same experienced radiologist while the participants were in centric occlusion and a standardized upright position, with the transporionic axis and Frankfort horizontal plane parallel to the surface of the floor [32, 34]. A well-trained assessor (S. Elayah) used DOLPHIN Imaging Software (Dolphin Imaging Version 11.95.07.24 Premium, Chatsworth) [35] to trace twice within a 2-week interval to eliminate measurement errors [36, 37]. All the study variables were measured using stable landmarks, including 12 linear (mm) and 10 angular (º) variants. On each lateral cephalogram, the following landmarks were identified:

- Cranial Base; Anterior Cranial Base length, S–N (mm); Posterior Cranial Base length, S-Ba (mm) and Cranial Base Angle, S–N-Ba (º) (Fig. S1).
- Maxilla; Maxillary Length, Co-A (mm); Anterior Upper Facial Height, N-ANS (mm); Posterior Upper Facial Height, S- PM (mm); Maxillary Sagittal Position, SNA (º) and
- Maxillary anteroposterior inclination, SN-PP(º) (Fig. S2).
- Mandible: Mandibular length, Co-Gn (mm); Corpus (Body) Length, Go-Gn (mm); Ramus Height, Ar-Go (mm); Mandibular sagittal Position, SNB (º); Total Anterior Facial Height, N-Me (mm); Lower Anterior Facial height, ANS-Me(mm); Total Posterior Facial Height, S-Go (mm) and Mandible anteroposterior inclination, MP-SN(º) (Fig. S3).
- Mandibular Anteroposterior Inclination; Maxillomandibular differences, Co-Gn—Co-A(mm); Sagittal Intermaxillary Relationship, ANB (º); and Palatal-Mandibular Angle, PP-MP (º) (Fig. S4).
- Occlusion; Occlusal Plane to SN Plane, OP-SN (º); Occlusal Plane to FH Plane, OP-FH (º) and Occlusal Plane to Mandibular Plane, OP-MP (º) (Fig. S5).

### Statistical analysis

Social Sciences (SPSS) version 27, (Chicago, USA) used to perform computations for both descriptive and analytical statistics. The normality distribution of the data was assessed using the Kolmogorov–Smirnov test (Table S1). To evaluate variances in craniofacial morphology among the three groups, we employed the Kruskal–Wallis H, Mann–Whitney tests and independent *t-*test. Furthermore, we assessed the intra-examiner reliability of measurements using the intraclass correlation coefficient test (ICC). As well as the cephalometric measurement errors were measured using the Dahlberg formula [38]. Additionally, we considered a significance level of *P* < 0.05.

## Results

In this study, a total of 90 participants were involved, consisting of 60 patients with non-syndromic isolated cleft palate who underwent surgical repair using the S.F^+R.I^ technique (30) and S.F^−R.I^ technique (30) with no significant difference found between them regarding cleft type, cleft width, and age at repair. While the other 30 were normal participants with skeletal class I pattern, with no significant difference found among groups regarding gender and age at cephalogram collection (Table 1). The average ages at cephalogram collection were 6.03 ± 0.80 in the S.F^−R.I^ group, 5.96 ± 0.76 in the S.F^+R.I^ group, and 5.91 ± 0.87 in the control group (ranging from 5 to 7 in all groups). Furthermore, a comparison of maxillofacial morphology among the three groups was conducted and the results are presented in (Table 2). The ICC values for all metrics exceeded 0.95, indicating a satisfactory level of agreement. As well as the mean linear and the mean angular measurement errors were near the ideal value of zero (Table 3).

**Table 2:** Results of comparison of maxillofacial morphology between three groups using the Independent Samples (*t-*test) test (^*^) and the Mann–Whitney test (^#^)

Variables	S.F^+R.I^ group (I)	S.F^−R.I^ group (II)	Control group (**III**)	*P*-value		
	Mean ± SD	Mean ± SD	Mean ± SD	**I** vs **II I** vs **III II** vs **III**		
**Cranial base**						
S-N^a^	54.5 ± 4.5	55.4 ± 4.3	56.7 ± 4.3	0.46^*^	0.06^*^	0.23^*^
S-Ba^a^	29.4 ± 3.8	32.2 ± 4.1	32.6 ± 4.5	0.01^*^	< 0.01^*^	0.72^*^
S–N-Ba^b^	130.2 ± 5.3	128.8 ± 6.7	129.4 ± 5.3	0.39^*^	0.58^*^	0.71^*^
**Maxilla**						
Co-A^a^	57 ± 5.8	62.3 ± 5.4	65.1 ± 6.3	< 0.01^*^	< 0.01^*^	0.08^*^
N-ANS^a^	43.2 ± 4.3	42.4 ± 4.6	43.0 ± 4.0	0.50^*^	0.87^*^	0.6^*^
S- PM^a^	30.1 ± 2.7	31.1 ± 4.3	32.6 ± 4.0	0.29^*^	< 0.01^#^	0.07^#^
SNA^b^	74.2 ± 5	78.3 ± 5.0	79.4 ± 4.6	< 0.01^*^	< 0.01^*^	0.37^*^
SN-PP^b^	21.4 ± 5.2	19.6 ± 5.5	17.9 ± 4.2	0.22^*^	0.01^*^	0.18^*^
**Mandible**						
Co-Gn^a^	82.2 ± 7.2	83.0 ± 7.4	85.9 ± 8.2	0.76^#^	0.07^*^	0.16^*^
Go-Gn^a^	62.6 ± 7.2	60.8 ± 6.9	62.6 ± 6.8	0.38^#^	0.87^#^	0.30^*^
Ar-Go^a^	31.7 ± 2.9	32.5 ± 3.9	33.5 ± 4.1	0.36^*^	0.06^*^	0.35^*^
SNB^b^	75.1 ± 2.9	75.3 ± 5.2	76.2 ± 4.5	0.91^*^	0.38^*^	0.47^*^
N-Me^a^	95.9 ± 5.7	93.4 ± 13.3	96.7 ± 7.1	0.36^#^	0.77^#^	0.33^#^
ANS-Me^a^	52.7 ± 3	51.0 ± 11.9	53.7 ± 4.4	0.62^#^	0.32^*^	0.73^*^
S-Go^a^	57.7 ± 4.7	56.4 ± 8.7	59.1 ± 6.3	0.81^#^	0.22^#^	0.26^*^
MP-SN^b^	42 ± 5.5	42.0 ± 8.3	40.0 ± 6.8	0.58^#^	0.16^#^	0.48^#^
**Intermaxillary relation**						
Co-Gn—Co-A^a^	25.1 ± 3.8	20.1 ± 5.3	20.9 ± 3.5	< 0.01^*^	< 0.01^*^	0.48^*^
ANB^b^	-0.9 ± 3.6	3.1 ± 1.9	3.3 ± 1.3	< 0.01^*^	< 0.01^*^	0.64^*^
PP-MP^b^	20.6 ± 5.2	21.4 ± 5.2	22.1 ± 6.1	0.99^#^	0.43^#^	0.63^*^
**Occlusion**						
OP-SN^b^	21.9 ± 5.8	21.0 ± 6.2	20.0 ± 4.4	0.56^*^	0.16^*^	0.49^*^
OP-FH^b^	13.4 ± 4.5	13.4 ± 7.0	12.0 ± 5.1	0.98^*^	0.24^*^	0.36^*^
OP-MP^b^	20.1 ± 4.7	20.1 ± 7.2	20.0 ± 5.7	0.99^*^	0.96^*^	0.97^*^

*Abbreviations*: *S.F*^+*R.I*^ Sommerlad-Furlow modified technique with relaxing incisions, *S.F*^*−R.I*^ Sommerlad-Furlow modified technique without relaxing incisions, *S* sella, *N* nasion, *Ba* Basion, *Co* condylion, *A* A point, *ANS* anterior nasal spine, *PM* pterygomaxillare, *PP* palatal plane, *Gn* Gnathion, *Go* gonion, *B* B point, *Me* menton, *Ar* articular, *MP* Mandibular Plane, *OP* Occlusal Plane, *FH* Frankfort horizontal plane, *SD* standard deviation

^a^Distances between two landmarks were measured in millimeters (mm)

^b^Angles formed by three landmarks were measured in degrees (º)

Significant at the *p* < 0.05 level. Highly significant at the *p* = 0.01 level

^*^Independent Samples (*t-*test) Test

^#^Mann–Whitney Test

**Table 3:** The cephalometric measurement errors using the Dahlberg formula

Variables	S.F^+R.I^ Group (**I**)	S.F^−R.I^ Group (**II**)	Control Group (**III**)
	The cephalometric measurement errors		
**Cranial base**			
S–N	0.040	0.021	0.070
S-Ba	0.0012	0.059	0.042
S–N-Ba	0.025	0.044	0.012
**Maxilla**			
Co-A	0.064	0.012	0.038
N-ANS	0.046	0.036	0.038
S- PM	0.012	0.016	0.007
SNA	0.027	0.090	0.027
SN-PP	0.025	0.013	0.038
**Mandible**			
Co-Gn	0.023	0.099	0.025
Go-Gn	0.070	0.051	0.051
Ar-Go	0.033	0.071	0.053
SNB	0.014	0.056	0.044
N-Me	0.084	0.032	0.025
ANS-Me	0.044	0.039	0.012
S-Go	0.009	0.010	0.051
MP-SN	0.037	0.011	0.038
**Intermaxillary relation**			
Co-Gn—Co-A	0.071	0.061	0.012
ANB	0.009	0.003	0.005
PP-MP	0.009	0.042	0.016
**Occlusion**			
OP-SN	0.006	0.037	0.012
OP-FH	0.005	0.015	0.033
OP-MP	0.006	0.067	0.044

*Abbreviations*: S.F^+*R.I*^ Sommerlad-Furlow modified technique with relaxing incisions, *S.F*^*−R.I*^ Sommerlad-Furlow modified technique without relaxing incisions, *S* sella, *N* nasion, *Ba* Basion, *Co* condylion, *A* A point, *ANS* anterior nasal spine, *PM* pterygomaxillare, *PP* palatal plane, *Gn* Gnathion, *Go* gonion, *B* B point, *Me* menton, *Ar* articular, *MP* Mandibular Plane, *OP* Occlusal Plane, *FH* Frankfort horizontal plane

Regarding cranial base, the results showed that there were no statistically significant differences between the three groups (S.F^+R.I^, S.F^−R.I^ & C groups) in (S–N 54.5 ± 4.5, 55.4 ± 4.3 & 56.7 ± 4.3 and S–N-Ba; 130.2 ± 5.3, 128.8 ± 6.7 & 129.4 ± 5.3) respectively. While the S.F^+R.I^ group had a significantly shortest S-Ba than the S.F^−R.I^ & C groups (*P* = 0.01 & *P* < 0.01), but there was no statistically significant difference between S.F^−R.I^ & C groups (*P* = 0.72).

Regarding skeletal maxilla, there was no significant difference between the S.F^+R.I^ and S.F^−R.I^ groups in all linear measurements (N-ANS and S- PM) except Co-A, the S.F^+R.I^ group had significantly shorter Co-A than the S.F^−R.I^ & C groups (*P* =  < 0.01). While the angular measurement, S.F^+R.I^ group had significantly less SNA angle than the S.F^−R.I^ & C groups (*P* =  < 0.01).

Regarding mandibular bone, there were no statistically significant differences in all linear and angular mandibular measurements between the S.F^+R.I^ and S.F^−R.^groups. While S.F^+R.I^ group had slightly shorter Co-Gn and Ar-Go than the C group.

Regarding intermaxillary relation, the S.F^+R.I^ group had significant differences in Co-Gn—Co-A and ANB as compared with the S.F^−R.I^ & C groups (*P* =  < 0.01). While there was no statistically significant difference in PP-MP between the three groups.

Regarding occlusion, there were no significant differences in all angular occlusal measurements between the three groups.

## Discussion

The impact of relaxing incisions on maxillofacial growth during palatoplasty is still not fully understood and confusing evidence has been published [39]. However, our previous study which compared between S.F^+R.I^ and S.F^−R.I^ techniques in terms of Oronasal fistula, Velopharyngeal insufficiency, and Inadequate quality of life; concluded that there was a non-significant difference between S.F^+R.I^ and S.F^−R.I^ groups [24].

Thus, this study was aimed to estimate the impact of relaxing incisions on maxillofacial growth following S.F technique in patients with isolated cleft palate.

Our findings indicate that there were no statistically significant differences observed among the three groups (S.F^+R.I^, S.F^−R.I^ & C groups) in the anterior cranial base length and angle values. While the S.F^+R.I^ group had a significantly shortest posterior cranial base than the S.F^−R.I^ & C groups (*P* = 0.01 & *P* < 0.01). Liao et al. [40] reported that the stage of palate repair had a significant effect on the means of the length of the posterior cranial base (S-Ba)(*p* = 0.05). A clinical study [41] evaluated the application of buccal fat pads in pack palate relaxing incisions, concluded the control group (with iodoform gauze) showed significantly shortened cranial basal lengths (N-Ba) (*P* < 0.05). As well as, a systematic review concluded that the posterior cranial base is not totally stable, as its dimensions change throughout craniofacial growth and a minor dimensional change is observed even in late adulthood [42]. Also, some studies has postulated that the decreased cranial base length observed in individuals with BCLP might be linked to growth stunting during their early years, followed by a compensatory growth spurt in the later stages of development [35]. Koberg and Koblin [6] found that Veau's method of pushback and Langenbeck's technique involving relaxing incisions had the most adverse impact on the maxillofacial growth of patients with cleft palate. On the other hand, some studies reported that it appears improbable that mending the palate repair have any impact on the cranial base growth, because of its distance from the surgical site. One potential reason for this disparity could be variations in body height, which is related to the length of the cranial base [43, 44]. Thus, palatoplasty with relaxing incisions might not have an effect on posterior cranial base growth.

Most of studies did not specifically focus on the cranial base, but the maxilla is a key component of the cranial base. While comparing the measures of maxilla, we did not find significant differences between the S.F^+R.I^ and S.F^−R.I^ groups in all linear measurements except maxillary length which was significantly shorter Co-A than the S.F^−R.I^ group(*P* =  < 0.01). While, the relative anteroposterior relation of the maxilla to the cranial base, S.F^+R.I^ group had significantly less SNA angle than the S.F^−R.I^ & C groups (*P* =  < 0.01).

A randomized clinical trial study was conducted to investigate the impact of relaxing incisions on maxillary growth in individuals undergoing the two-flap and one-flap techniques. It concluded that there is no association between the implementation of relaxing incisions and any subsequent disruptions in maxillary growth [20]. This study failed to offer conclusive evidence on the association between the utilization of relaxing incisions and potential maxillary growth impairment due to the absence of a control group. Moreover, both trial groups employed relaxing incisions. In contrast, Tanino et al. [45] who compared between two different protocols for palatoplasty. In one group, a vomer flap was used, while in the other group, the repair was done by push-back technique with relaxing incisions. They concluded that the use of a vomer flap resulted in favorable maxillary growth. This was attributed to the fact that no relaxing incisions were made, avoiding secondary intention healing. On the other hand, the utilization of the minimal incision technique has demonstrated superior outcomes in the advancement of the maxilla [46]. This context in agreement with our results.

Some studies suggest that the utilization of surgical relaxing incisions during primary palatoplasty can have a notably adverse impact on maxillary growth [9–13]. On the contrary, several studies have found no correlation between the utilization of these incisions and growth impairment [20, 21, 47].

In term of mandible, there was no significant difference in all linear and angular measurements of mandible in the S.F^+R.I^ and S.F^−R.I^ groups. Our results are consistent with previous studies, which found that the technique of hard palate repair had no significant impact on either the mandibular plane inclination or the mandibular protrusion [48–50]. Shibasaki and Ross [51] reported the mandible is of normal length but retropositioned due to the functional response of the mandible to the altered maxilla. Thus, our results of mandibular measurements may explain that maxillary growth was satisfactory growth which obtained with both techniques.

In term of maxillo-mandibular relationship, our results showed that the S.F^+R.I^ group had significant differences in Co-Gn—Co-A and ANB as compared with the S.F^−R.I^ & C groups (*P* =  < 0.01). While there was no significant differences between the S.F^−R.I^ & C groups in the intermaxillary relationship. Da Silva et al. [52] the intermaxillary relationship was regarded as satisfactory, and the facial pattern did not affect the primary palatoplasty. Some studies [48–50] reported that the palatoplasty did not significantly affect jaw relation (ANB). The technique may result in more palatal scar tissue, which might have a greater impact on the alveolar process and teeth than on maxillary growth [50]. On the other hand, incisor relations and articulation were enhanced by a decrease in periosteal undermining and a reduction of the palatal region, which was left exposed following palatal repair [53]. Similarly, when compared to the Veau-Wardill-Kilner technique, which was reported to create relatively large regions of denuded palatal bone, the minimal incision technique contributed to improved maxilla growth and dental occlusion [46].

In term of Occlusion, an experiment study showed that mucoperiosteal denudation of the palate had a greater effect on the inclination of the teeth [54]. Another study has been compared the dental occlusion in two techniques repair of isolated clefts of the hard and soft palate, reported that the minimal incision technique has been shown to result in better a better dental occlusion and palatal mucosa with significantly less scar tissue [46]. These studies in consistent with our results, which showed a satisfactory dental occlusion in S.F^−R.I^ group, but they interpret our results of S.F^+R.I^ group, there was no significant difference in all angular measurements in three groups. Odom et al. [21] found no relationship between the kind of palatal incisions created during the closure of an isolated cleft palate and the subsequent formation of a class III incisal relation.

Overall, the lateral relaxing incisions remain a probable factor among the other possible causes for maxillary growth disruption following cleft palate surgery, while there is no consensus in the literature about the causal independent factor for this condition [20, 47]. The current favorable outcomes observed in both primary palatoplasty techniques may be clarified through the conclusions of two systematic review studies; It is generally accepted that cleft lip repair may have a negative impact on maxillofacial growth; thus, lip closure is the most significant factor in inhibiting maxillary growth in those with UCLP [55, 56]. However, the tension generated by upper lip closure results in retro-inclined upper incisors, a retruded maxilla, and an obtuse nasolabial angle [57]. Typically, this results in an anterior crossbite [58].

The favorable outcomes observed in the S-F technique may be attributed to the three concepts that the S-F technique designed to close the cleft palate under palatal muscle reconstruction using Sommerlad muscle dissection, decreasing the pharyngeal cavity by nasal Z-plasty and a novel incision on the medial pterygoid plate's surface which was designed to make the S-F technique applicable in wider clefts without relaxing incision on the hard palate [23].

The outcomes associated with this study may have been impacted by its limitations. The groups were assessed before puberty. As well as, it did not assess the postoperative complications, including oronasal fistula, and velopharyngeal insufficiency. However, our previous study which compared between S.F^+R.I^ and S.F^−R.I^ technique in terms of Oronasal fistula, Velopharyngeal insufficiency, and Inadequate quality of life, concluded that there was a non-significant difference between the relaxing incision (S.F^+R.I^) and non-relaxing incision (S.F^−R.I^) groups [24]. Another limitation was that the patients included were not all from the same surgeon. However, in this study, both surgeons had more than 12 years of experience and worked in almost the same team. Further evaluation of prospective study with a large size would be warranted.

## Conclusion

As a preliminary report, the Sommerlad-Furlow modified technique without relaxing incisions was found to have good maxillary positioning in the face and satisfactory intermaxillary relationship compared to the Sommerlad-Furlow modified technique with relaxing incisions. Thus, there seems that the use of relaxing incisions may be related to maxillary growth impairment in patients aged 5–7 years. However, longer-term studies are needed to confirm these results.

### Supplementary Information

**Additional file 1: Fig. S1.** Cranial Base measurements; Anterior cranial base length (S-N, Sella-Nasion); Posterior cranial base length (S-Ba, Sella- Basion); Cranial base angle (S-N-Ba, Sella-Nasion-Basion angle). **Fig. S2.** Maxilla measurements; Maxillary Length (Co-A, condylion - A point); Anterior Upper Facial Height (N-ANS, Nasion- anterior nasal spine); Posterior Upper Facial Height (S- PM, Sella - pterygomaxillare); Sagittal Maxillary Position (SNA, Sella-Nasion- A point angle), and Maxillary Anteroposterior Inclination (SN-PP, Sella-Nasion line- palatal plane angle). **Fig. S3. **Mandible measurements; Mandibular Length (Co-Gn, condylion- Gnathion); Corpus (Body) Length (Go-Gn, gonion -Gnathion); Ramus Height (Ar-Go, articular- gonion); Mandibular sagittal Position (SNB, Sella-Nasion- B point angle); Total Anterior Facial Height (N-Me, Nasion- mention); Lower Anterior Facial Height (ANS-Me, anterior nasal spine -mention), Posterior Total Facial Height (S-Go, Sella- gonion) and Mandibular Anteroposterior Inclination (MP – SN, mandibular plane- Sella Nasion line angle). **Fig. S4.** Intermaxillary relation measurements; Maxillo-mandibular differences (Co-Gn - Co-A, condylion- Gnathion- condylion - articular); Sagittal intermaxillary relationship (ANB, A point -Nasion - B point angle) and Palatal plane - mandibular plane (PP-MP,) angle. **Fig. S5.** Occlusion measurements; Occlusal plane to anterior cranial base angle (OP-SN, Occlusal plane- Sella Nasion line angle); Occlusal Plane to Frankfort horizontal plane angle (OP-FH) angle, and Occlusal plane to mandibular plane (OP-MP) angle.

**Additional file 2: Table S1.** Comparison of data distribution between three groups using the Kolmogorov-Smirnov test.

## Data Availability

The datasets used and analyzed during the study are available from the corresponding author upon reasonable request.

## Abbreviations

S.F^+R.I^: Sommerlad-Furlow modified technique with relaxing incisions
S.F^−^^R.I^: Sommerlad-Furlow modified technique without relaxing incisions
S: Sella
N: Nasion
Ba: Basion
Co: Condylion
A: A point
ANS: Anterior nasal spine
PM: Pterygomaxillare
PP: Palatal plane
Gn: Gnathion
Go: Gonion
B: B point
Me: Menton
Ar: Articular
MP: Mandibular Plane
OP: Occlusal Plane
FH: Frankfort horizontal plane
SD: Standard deviation
(mm): Distances between two landmarks were measured in millimeters
(º): Angles formed by three landmarks were measured in degrees
