# Supplementary material for: Impact of relaxing incisions on maxillofacial growth following Sommerlad–Furlow modified technique in patients with isolated cleft palate: a preliminary comparative study
Source: BMC Surg. 2023 Nov 23;23:358. doi: 10.1186/s12893-023-02247-5 (PMC10668437; doi:10.1186/s12893-023-02247-5)

**Supplementary figures legend**

**Fig. S1.** **Cranial Base measurements;** Anterior cranial base length (S-N, Sella-Nasion); Posterior cranial base length (S-Ba, Sella- Basion); Cranial base angle (S-N-Ba, Sella-Nasion-Basion angle).


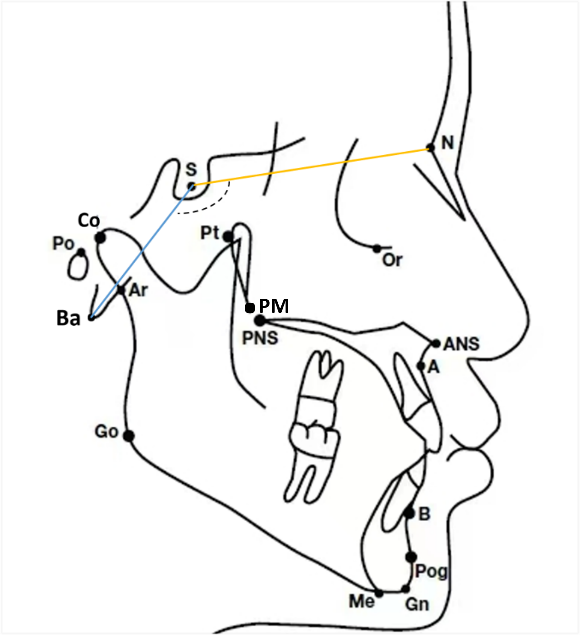


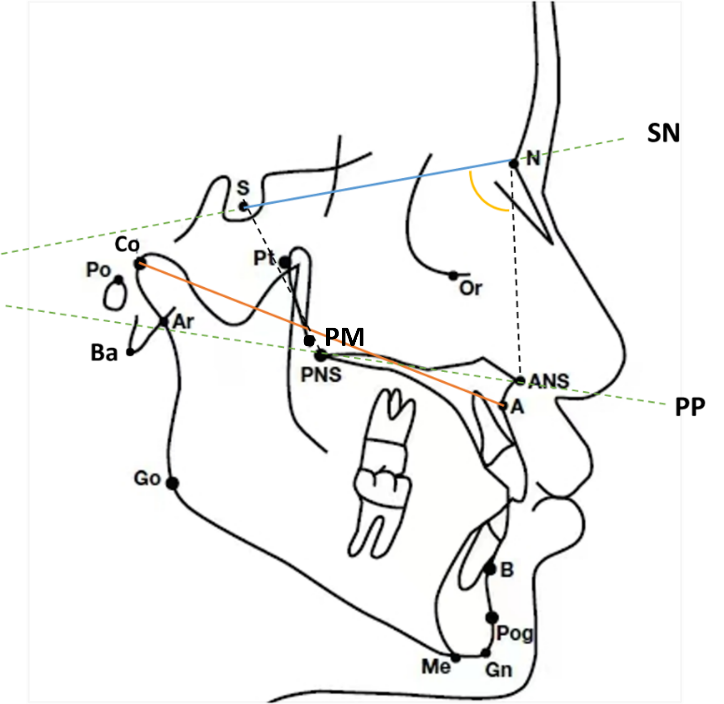
**Fig. S2. Maxilla measurements;** Maxillary Length (Co-A, condylion - A point); Anterior Upper Facial Height (N-ANS, Nasion- anterior nasal spine); Posterior Upper Facial Height (S- PM, Sella - pterygomaxillare); Sagittal Maxillary Position (SNA, Sella-Nasion- A point angle), and Maxillary Anteroposterior Inclination (SN-PP, Sella-Nasion line- palatal plane angle).

**Fig. S3. Mandible measurements;** Mandibular Length (Co-Gn, condylion- Gnathion); Corpus (Body) Length (Go-Gn, gonion -Gnathion); Ramus Height (Ar-Go, articular- gonion); Mandibular sagittal Position (SNB, Sella-Nasion- B point angle); Total Anterior Facial Height (N-Me, Nasion- mention); Lower Anterior Facial Height (ANS-Me, anterior nasal spine -mention), Posterior Total Facial Height (S-Go, Sella- gonion) and Mandibular Anteroposterior Inclination (MP – SN, mandibular plane- Sella Nasion line angle).


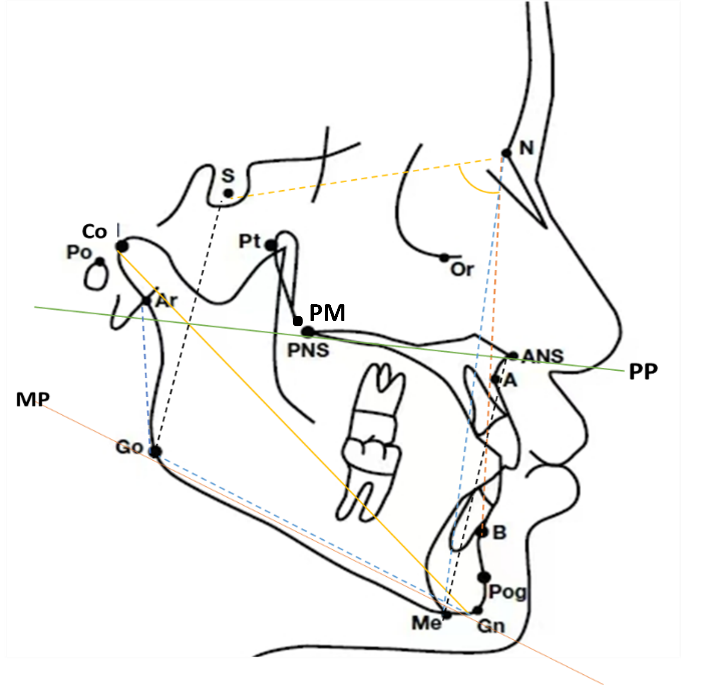


**Fig. S4. Intermaxillary relation measurements;** Maxillo-mandibular differences (Co-Gn - Co-A, condylion- Gnathion- condylion - articular); Sagittal intermaxillary relationship (ANB, A point -Nasion - B point angle) and Palatal plane - mandibular plane (PP-MP,) angle.


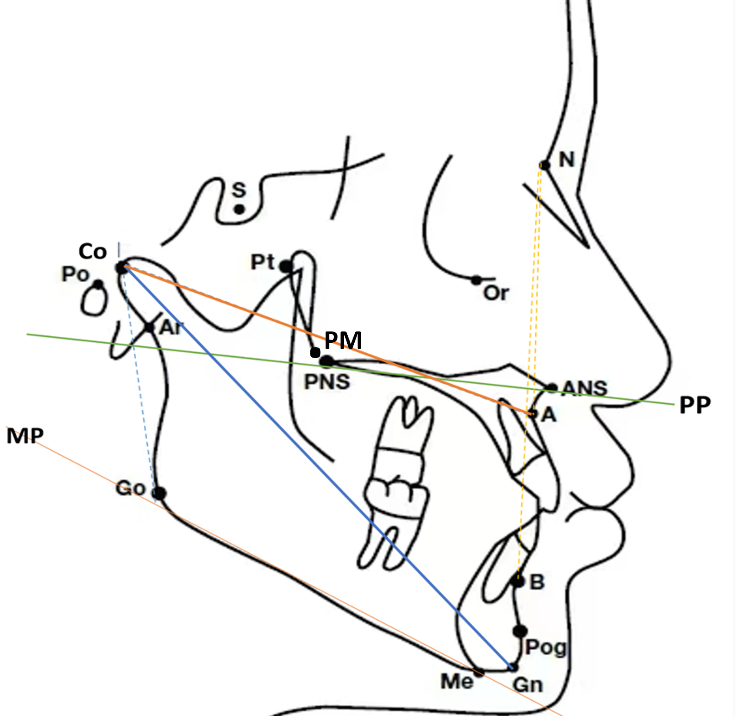


**Fig. S5. Occlusion measurements;** Occlusal plane to anterior cranial base angle (OP-SN, Occlusal plane- Sella Nasion line angle); Occlusal Plane to Frankfort horizontal plane angle (OP-FH) angle, and Occlusal plane to mandibular plane (OP-MP) angle.


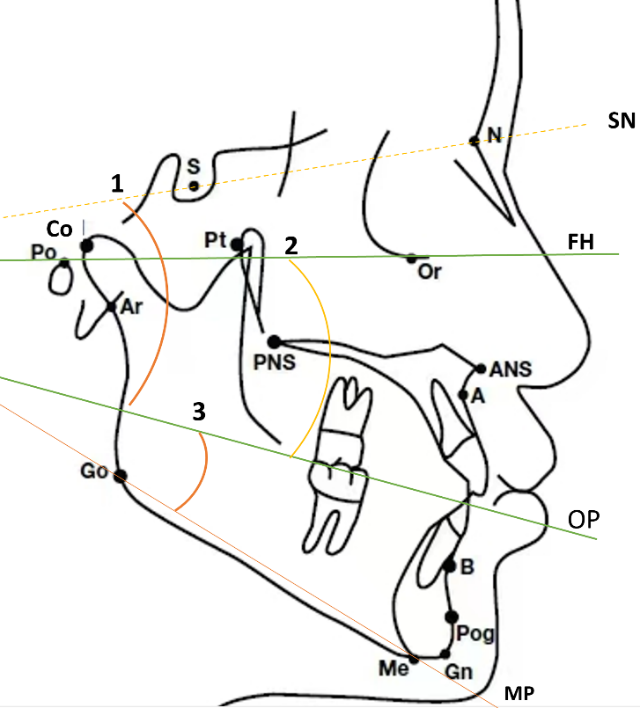

Supplement: Supplementary file 1 — Additional file 1: Fig. S1. Cranial Base measurements; Anterior cranial base length (S-N, Sella-Nasion); Posterior cranial base length (S-Ba, Sella- Basion); Cranial base angle (S-N-Ba, Sella-Nasion-Basion angle). Fig. S2. Maxilla measurements; Maxillary Length (Co-A, condylion - A point); Anterior Upper Facial Height (N-ANS, Nasion- anterior nasal spine); Posterior Upper Facial Height (S- PM, Sella - pterygomaxillare); Sagittal Maxillary Position (SNA, Sella-Nasion- A point angle), and Maxillary Anteroposterior Inclination (SN-PP, Sella-Nasion line- palatal plane angle). Fig. S3. Mandible measurements; Mandibular Length (Co-Gn, condylion- Gnathion); Corpus (Body) Length (Go-Gn, gonion -Gnathion); Ramus Height (Ar-Go, articular- gonion); Mandibular sagittal Position (SNB, Sella-Nasion- B point angle); Total Anterior Facial Height (N-Me, Nasion- mention); Lower Anterior Facial Height (ANS-Me, anterior nasal spine -mention), Posterior Total Facial Height (S-Go, Sella- gonion) and Mandibular Anteroposterior Inclination (MP – SN, mandibular plane- Sella Nasion line angle). Fig. S4. Intermaxillary relation measurements; Maxillo-mandibular differences (Co-Gn - Co-A, condylion- Gnathion- condylion - articular); Sagittal intermaxillary relationship (ANB, A point -Nasion - B point angle) and Palatal plane - mandibular plane (PP-MP,) angle. Fig. S5. Occlusion measurements; Occlusal plane to anterior cranial base angle (OP-SN, Occlusal plane- Sella Nasion line angle); Occlusal Plane to Frankfort horizontal plane angle (OP-FH) angle, and Occlusal plane to mandibular plane (OP-MP) angle. [file 12893_2023_2247_MOESM1_ESM.docx]
