# Supplementary material for: Impact of relaxing incisions on maxillofacial growth following Sommerlad–Furlow modified technique in patients with isolated cleft palate: a preliminary comparative study
Source: BMC Surg. 2023 Nov 23;23:358. doi: 10.1186/s12893-023-02247-5 (PMC10668437; doi:10.1186/s12893-023-02247-5)
Supplement: Supplementary file 2 — Additional file 2: Table S1. Comparison of data distribution between three groups using the Kolmogorov-Smirnov test. [file 12893_2023_2247_MOESM2_ESM.docx]

**Table S1.** Comparison of data distribution between three groups using the Kolmogorov-Smirnov test.

| Variables | **S.F^+R.I^ vs S.F^-R.I^**  **groups** | **S.F^+R.I^ vs Control**  **groups** | **S.F^-R.I^ vs Control**  **groups** |
| --- | --- | --- | --- |
| **Cranial Base** |  |  |  |
| S-N^a^ | Normal distribution.  Independent Samples (*t*) test | Normal distribution.  Independent Samples (*t*) test | Normal distribution.  Independent Samples (*t*) test |
| S-Ba^a^ | Normal distribution.  Independent Samples (*t*) test | Normal distribution.  Independent Samples (*t*) test | Normal distribution.  Independent Samples (*t*) test |
| S-N-Ba^b^ | Normal distribution.  Independent Samples (*t*) test | Normal distribution.  Independent Samples (*t*) test | Normal distribution.  Independent Samples (*t*) test |
| **Maxilla** |  |  |  |
| Co-A^a^ | Normal distribution.  Independent Samples (*t*) test | Normal distribution.  Independent Samples (*t*) test | Normal distribution.  Independent Samples (*t*) test |
| N-ANS^a^ | Normal distribution.  Independent Samples (*t*) test | Normal distribution.  Independent Samples (*t*) test | Normal distribution.  Independent Samples (*t*) test |
| S- PM^a^ | Normal distribution.  Independent Samples (*t*) test | Non-Normal distribution.  Mann-Whitney test | Non-Normal distribution.  Mann-Whitney test |
| SNA^b^ | Normal distribution.  Independent Samples (*t*) test | Normal distribution.  Independent Samples (*t*) test | Normal distribution.  Independent Samples (*t*) test |
| SN-PP^b^ | Normal distribution.  Independent Samples (*t*) test | Normal distribution.  Independent Samples (*t*) test | Normal distribution.  Independent Samples (*t*) test |
| **Mandible** |  |  |  |
| Co-Gn^a^ | Non-Normal distribution.  Mann-Whitney test | Normal distribution.  Independent Samples (*t*) test | Normal distribution.  Independent Samples (*t*) test |
| Go-Gn^a^ | Non-Normal distribution.  Mann-Whitney test | Non-Normal distribution.  Mann-Whitney test | Normal distribution.  Independent Samples (*t*) test |
| Ar-Go^a^ | Normal distribution.  Independent Samples (*t*) test | Normal distribution.  Independent Samples (*t*) test | Normal distribution.  Independent Samples (*t*) test |
| SNB^b^ | Normal distribution.  Independent Samples (*t*) test | Normal distribution.  Independent Samples (*t*) test | Normal distribution.  Independent Samples (*t*) test |
| N-Me^a^ | Non-Normal distribution.  Mann-Whitney test | Non-Normal distribution.  Mann-Whitney test | Non-Normal distribution.  Mann-Whitney test |
| ANS-Me^a^ | Non-Normal distribution.  Mann-Whitney test | Normal distribution.  Independent Samples (*t*) test | Normal distribution.  Independent Samples (*t*) test |
| S-Go^a^ | Non-Normal distribution.  Mann-Whitney test | Normal distribution.  Independent Samples (*t*) test | Normal distribution.  Independent Samples (*t*) test |
| MP-SN^b^ | Non-Normal distribution.  Mann-Whitney test | Non-Normal distribution.  Mann-Whitney test | Non-Normal distribution.  Mann-Whitney test |
| **Intermaxillary relation** |  |  |  |
| Co-Gn - Co-A^a^ | Normal distribution.  Independent Samples (*t*) test | Normal distribution.  Independent Samples (*t*) test | Normal distribution.  Independent Samples (*t*) test |
| ANB^b^ | Normal distribution.  Independent Samples (*t*) test | Normal distribution.  Independent Samples (*t*) test | Normal distribution.  Independent Samples (*t*) test |
| PP-MP^b^ | Non-Normal distribution.  Mann-Whitney test | Non-Normal distribution.  Mann-Whitney test | Normal distribution.  Independent Samples (*t*) test |
| **Occlusion** |  |  |  |
| OP-SN^b^ | Normal distribution.  Independent Samples (*t*) test | Normal distribution.  Independent Samples (*t*) test | Normal distribution.  Independent Samples (*t*) test |
| OP-FH^b^ | Normal distribution.  Independent Samples (*t*) test | Normal distribution.  Independent Samples (*t*) test | Normal distribution.  Independent Samples (*t*) test |
| OP-MP^b^ | Normal distribution.  Independent Samples (*t*) test | Normal distribution.  Independent Samples (*t*) test | Normal distribution.  Independent Samples (*t*) test |

**Abbreviations**: S.F^+R.I^; Sommerlad-Furlow modified technique with relaxing incisions. S.F^-R.I^; Sommerlad-Furlow modified technique without relaxing incisions., S, sella; N, nasion; Ba, Basion; Co, condylion; A, A point; ANS, anterior nasal spine; PM, pterygomaxillare; PP, palatal plane; Gn, Gnathion; Go, gonion; B, B point; Me, menton; Ar, articular; MP, Mandibular Plane; OP, Occlusal Plane; FH, Frankfort horizontal plane; SD, standard deviation;

^a^ Distances between two landmarks were measured in millimeters (mm).

^b^ Angles formed by three landmarks were measured in degrees (º).

Signiﬁcant at the p < 0.05 level. Highly signiﬁcant at the p = 0.01 level.
